# Blastocystis and chronic urticaria: the evidence reviewed does not justify routine testing or treatment

**DOI:** 10.1099/jmm.0.002167

**Published:** 2026-05-11

**Authors:** Anastasios D. Tsaousis, Eleni Gentekaki, Funda Dogruman Al

**Affiliations:** 1 Laboratory of Molecular and Evolutionary Parasitology, School of Natural Sciences, University of Kent, Canterbury, Kent, UK; 2 Department of Veterinary Medicine, University of Nicosia School of Veterinary Medicine, Nicosia, Cyprus; 3 Division of Medical Parasitology, Department of Medical Microbiology, Faculty of Medicine, Gazi University, Ankara, Turkey

**Keywords:** *Blastocystis*, gut microbiome, parasite, urticaria

## Abstract

Ulusan Bagci *et al*. propose *Blastocystis* spp. as a hidden cause of chronic urticaria and suggest routine stool testing and treatment in positive cases. We argue that this conclusion is not supported by the current evidence, which is dominated by heterogeneous diagnostics, cross-sectional prevalence comparisons prone to misclassification and non-randomized treatment-response reports lacking clearance-linked endpoints. We outline minimum standards for interpretable inference, including quantitative detection, longitudinal sampling and trials linking confirmed clearance vs. persistence to validated urticaria outcomes.

## Introduction

Ulusan Bagci and colleagues review *Blastocystis* spp. as ‘the potential hidden cause of chronic urticaria’ and conclude that parasitological stool examination and treatment of *Blastocystis*-positive patients ‘may be beneficial’ [1]. This is a clinically actionable recommendation, but a critical appraisal of the literature does not support it. The review argues that, in chronic urticaria of uncertain cause, routine stool parasitology should be performed because identifying *Blastocystis* may reveal a treatable aetiology. However, in the absence of standardized, quantitative detection (e.g. quantitative PCR for load estimation with quality control) and without longitudinal sampling to limit misclassification, a single cross-sectional ‘positive’ result cannot be interpreted as evidence for disease-relevant exposure and should not justify routine screening. The review further implies that treatment of *Blastocystis*-positive patients may be beneficial and presents clinical improvement after metronidazole treatment as support for causality [1]. In chronic spontaneous urticaria (CSU), symptomatic improvement should be attributable to *Blastocystis* only if improvement is linked to confirmed organism clearance vs. persistence. Clearance should be established using quantitative assays, ideally with subtype/allele confirmation. Concurrent urticaria therapy (e.g. antihistamines, omalizumab) and detection of other possible aetiological agents should also be documented; without these, an antibiotic-associated response cannot be interpreted as an organism-specific cause.

### Diagnostic heterogeneity makes the prevalence signal unstable

The review acknowledges major sensitivity differences across microscopy, culture and PCR [1]. Yet, it pools prevalence ranges in CSU (21.3–61.1%) vs. controls (8–29.41%) as though these studies were methodologically comparable [1], when in fact this is not the case. Pooling data across heterogeneous methods of detection, variable sampling intensity and positivity thresholds produce systematic detection error that can inflate between-study variability and generate associations that are artefacts of methodology. This is particularly relevant for *Blastocystis*, where detectability can vary over time. Consistent with patterns observed across other gut protists, *Blastocystis* detectability may vary cyclically, suggesting that single-time-point testing is a poor basis for mechanistic inference or clinical action [2].

### Case reports do not justify ‘treat the positive’

Case reports describe improvement in urticaria after identifying and treating *Blastocystis* [e.g. 3, 4, 5]. These are useful signals, but they cannot separate the organism-driven effect from the natural ups and downs of a fluctuating disease, concurrent escalation of CSU therapy, co-pathogen effects or antibiotic-driven microbiome perturbation. Importantly, actionability is also limited by documented persistence and treatment failure. In a case series, Roberts *et al*. reported treatment failure and persistence of the same subtype despite multiple antimicrobial regimes in symptomatic individuals [6]. This undermines any implication that a positive result should routinely lead to eradication attempts, especially when the clinical endpoint is extra-intestinal and the causal chain is unproven.

### Subtype/amoeboid narratives are over-weighted relative to the strength of evidence

The review emphasizes Subtype 3 and ‘amoeboid forms’ are more pathogenic in urticaria. Reports linking amoeboid ST3 with urticaria presentations [e.g. 6,7] and emerging subtype/allele-level epidemiology suggesting enrichment of specific alleles in CSU cohorts (e.g. ST3 allele 34) [8,9] are noteworthy signals, and some are supporting immunological evidence for subtype-specific host responses. However, subtype/allele distributions are strongly population- and exposure-structured, and the CSU evidence remains methodologically heterogeneous and underpowered for clinical translation. These observations warrant better-designed studies; they do not yet justify routine testing or treatment recommendations based solely on subtype. Subtype/allele distributions are strongly population- and exposure-structured, and the CSU evidence remains methodologically heterogeneous and underpowered for translation in clinical settings.

### Microbiome arguments are not yet a basis for causality or treatment

The review links CSU to dysbiosis and implies *Blastocystis* itself (not the CSU–*Blastocystis* combination) may reduce diversity and beneficial taxa, thereby contributing indirectly to urticaria [1]. This claim sits in tension with a body of evidence associating *Blastocystis* with higher microbiome diversity in healthy individuals [10]. Even if one accepts the CSU dysbiosis literature, attributing it to *Blastocystis* requires data temporality, dose–response and reversibility tied to *Blastocystis* dynamics that the review does not provide. Moreover, antibiotic exposure confounds microbiome-mediated claims. Previously published longitudinal case work illustrates how antibiotic administration intersects with *Blastocystis* persistence and microbiome–metabolome trajectories, reinforcing why clinical response without clearance-linked microbiology is not interpretable as *Blastocystis*-specific [11].

## What should replace the review’s implied clinical pathway?

**Fig. 1.**
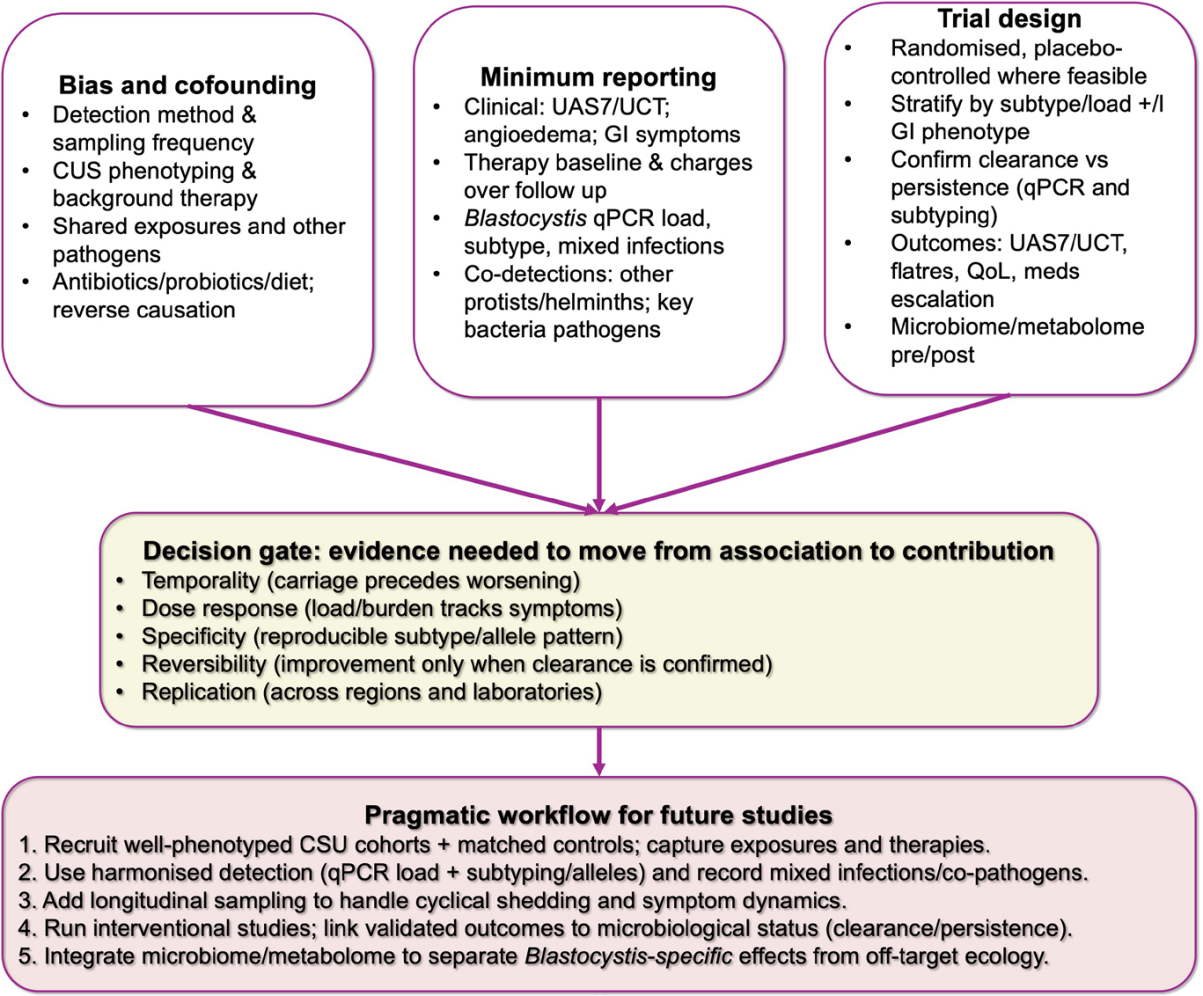
Causal-evidence roadmap linking *Blastocystis* carriage to chronic urticaria. Single-panel schematic outlining the minimum steps required to move from observational association to credible causal inference. The left panel highlights major threats to validity that commonly affect this literature, including exposure misclassification driven by diagnostic modality and sampling frequency, heterogeneity in CSU phenotyping and background therapy, shared environmental exposures, co-pathogens/co-colonizers, antibiotic/probiotic and diet-related microbiome shifts, and reverse causation. The middle panel lists minimum reporting items needed for interpretability across studies: standardized CSU outcomes (UAS7 or UCT), angioedema and gastrointestinal symptoms, documentation of concurrent treatments and changes over follow-up, harmonized *Blastocystis* reporting (method and quality control, quantitative load, subtype/allele assignment and mixed infections) and systematic co-detection of other enteric microbes. The right panel summarizes trial features capable of testing causality, emphasizing designs that stratify by load/subtype and relevant phenotypes, confirm clearance versus persistence using quantitative assays with typing and assess validated clinical endpoints (UAS7/UCT, flare frequency, quality of life and medication escalation), with parallel microbiome/metabolome profiling to separate *Blastocystis*-specific effects from off-target ecological changes. The ‘decision gate’ specifies the causal criteria required to argue for contribution rather than correlation: temporality, dose–response, specificity, reversibility, and linkage to confirmed clearance and replication across settings.

To make *Blastocystis* clinically interpretable in CSU, the minimum evidentiary logic must be explicit and standardized. We propose a compact roadmap (Fig. 1) that makes this practical:

(i) specify the dominant biases/confounders up front (assay choice, intermittent shedding, shared exposures, co-detections, concurrent urticaria therapy)

(ii) require minimum reporting standards (validated CSU outcomes such as UAS7/UCT; longitudinal sampling; quantitative detection; subtype/allele assignment; systematic co-detection of mixed infections and other enteric organisms; and prospective therapy tracking)

(iii) in interventional studies, treat confirmed clearance vs. persistence (quantitative assays with typing) as the mediator linked to validated CSU outcomes, ideally with parallel microbiome/metabolome profiling to distinguish organism-specific effects from off-target ecological change. This direction aligns with the ongoing One Health standardization aims within COST Action CA21105 [12].

## Conclusion

*Blastocystis* pathogenicity remains a plausible hypothesis in selected CSU contexts. Correlation does not establish causation: the criteria required for causal inference, temporality, dose–response, specificity and reversibility linked to confirmed clearance, have not yet been met in this literature. However, the evidence compiled in the reviewed article does not justify routine stool screening or antimicrobial treatment driven by *Blastocystis* detection alone. Clinical recommendations should be held to clearance-linked outcomes supported by quantitative detection and confounder-aware design.

## Abbreviations

CSU: chronic spontaneous urticaria
